# Improved exome prioritization of disease genes through cross-species phenotype comparison

**DOI:** 10.1101/gr.160325.113

**Published:** 2014-02

**Authors:** Peter N. Robinson, Sebastian Köhler, Anika Oellrich, Sanger Mouse Genetics Project, Kai Wang, Christopher J. Mungall, Suzanna E. Lewis, Nicole Washington, Sebastian Bauer, Dominik Seelow, Peter Krawitz, Christian Gilissen, Melissa Haendel, Damian Smedley

**Affiliations:** 1 Institute for Medical and Human Genetics, Charité-Universitätsmedizin Berlin, Augustenburger Platz 1, 13353 Berlin, Germany;; 2 Berlin Brandenburg Center for Regenerative Therapies, Charité-Universitätsmedizin Berlin, Augustenburger Platz 1, 13353 Berlin, Germany;; 3 Max Planck Institute for Molecular Genetics, 14195 Berlin, Germany;; 4 Mouse Informatics group, Wellcome Trust Sanger Institute, Hinxton, CB10 1SA, United Kingdom;; 5 Zilkha Neurogenetic Institute, University of Southern California, Los Angeles, California 90089, USA;; 6 Genomics Division, Lawrence Berkeley National Laboratory, Berkeley, California 94720, USA;; 7 Department of Neuropaediatrics Charité-Universitätsmedizin Berlin, Augustenburger Platz 1, 13353 Berlin, Germany;; 8 Department of Human Genetics, Nijmegen Centre for Molecular Life Sciences and Institute for Genetic and Metabolic Disorders, Radboud University Nijmegen Medical Centre, 6500 HB Nijmegen, The Netherlands;; 9 University Library and Department of Medical Informatics and Epidemiology, Oregon Health and Sciences University, Portland, Oregon 97239, USA

## Abstract

Numerous new disease-gene associations have been identified by whole-exome sequencing studies in the last few years. However, many cases remain unsolved due to the sheer number of candidate variants remaining after common filtering strategies such as removing low quality and common variants and those deemed unlikely to be pathogenic. The observation that each of our genomes contains about 100 genuine loss-of-function variants makes identification of the causative mutation problematic when using these strategies alone. We propose using the wealth of genotype to phenotype data that already exists from model organism studies to assess the potential impact of these exome variants. Here, we introduce PHenotypic Interpretation of Variants in Exomes (PHIVE), an algorithm that integrates the calculation of phenotype similarity between human diseases and genetically modified mouse models with evaluation of the variants according to allele frequency, pathogenicity, and mode of inheritance approaches in our Exomiser tool. Large-scale validation of PHIVE analysis using 100,000 exomes containing known mutations demonstrated a substantial improvement (up to 54.1-fold) over purely variant-based (frequency and pathogenicity) methods with the correct gene recalled as the top hit in up to 83% of samples, corresponding to an area under the ROC curve of >95%. We conclude that incorporation of phenotype data can play a vital role in translational bioinformatics and propose that exome sequencing projects should systematically capture clinical phenotypes to take advantage of the strategy presented here.

Whole-exome sequencing (WES) has revolutionized research into novel disease-gene discovery by enabling the inexpensive and rapid sequencing of nearly all human genes, with over 100 disease-gene identifications by WES since the first published success in 2010 (Ng et al. 2010b; Rabbani et al. 2012). Common bioinformatic analysis strategies for this data employ a series of filters designed to remove low quality and common variants and those deemed unlikely to be pathogenic (noncoding, not affecting splicing, synonymous or missense mutations annotated as nonpathogenic by prediction algorithms). Subsequently, the best candidates are chosen from among the remaining variants by strategies such as intersection of the results of WES from multiple individuals affected by the same disorder, linkage data or identity-by-descent inference, or by restricting the candidate list to genes of a certain pathway (Robinson et al. 2011). For instance, a number of the disease-gene discoveries reported to date have exploited the availability of multiple unrelated individuals with the same, clinically easily recognizable syndrome (Ng et al. 2010a) or the identification of de novo heterozygous mutations by trio analysis (Vissers et al. 2010). However, these approaches will not scale well for other classes of rare disease, including very rare disorders and dominant disorders in isolated small families.

One of the main challenges for disease-gene discovery by WES lies in the sheer number of variants found in individual exomes. An individual exome typically harbors over 30,000 variants compared with the genomic reference sequence, up to roughly 10,000 of which are predicted to lead to nonsynonymous amino acid substitutions, alterations of conserved splice site residues, or small insertions or deletions. Even after filtering out common variants, additional methods are needed to predict which variants may have serious functional consequences and prioritize them for validation (Pelak et al. 2010; Li et al. 2013). Methods exist to identify which variants deleteriously affect the function of individual proteins based only on characteristics such as conservation, physicochemical properties of the wild type and variant amino acids, and other protein features. However, each genome is thought to harbor about 100 genuine loss-of-function variants with about 20 genes completely inactivated (MacArthur et al. 2012). We therefore reasoned that prioritization based purely on sequence variant pathogenicity will struggle to correctly distinguish the disease-associated mutation from other variants with a deleterious biochemical effect.

A wealth of genotype to phenotype data already exists from model organism studies that can be used to assess the potential impact of these exome variants. For example, the Mouse Genome Informatics (MGI) database (Bult et al. 2013) currently contains phenotype annotations for some 8786 genes. Complementing this manual curation of community-wide publications, the International Mouse Phenotyping Consortium (IMPC; http://www.mousephenotype.org) is in the process of generating phenotype data for nearly all 20,000 protein-coding genes over the next decade, providing an unprecedented insight into mammalian gene function as well as a valuable resource for understanding human disease (Brown and Moore 2012). There are currently 4836 protein-coding human genes with a phenotyped mouse mutant of the ortholog (based on data from MGD downloaded 01/05/13; http://www.informatics.jax.org) but no known genotype to phenotype association from involvement in a Mendelian disease (based on data downloaded from OMIM 01/05/13; http://omim.org). To utilize this data, we have developed cross-species analysis approaches that allow computational reasoning with the Human Phenotype Ontology (HPO) (Robinson et al. 2008) and the Mammalian Phenotype Ontology (MPO) (Smith et al. 2005) to identify similarities between human disease manifestations and observations made in genetically modified model organisms (Washington et al. 2009; Mungall et al. 2010; Doelken et al. 2013; Köhler et al. 2013). These previous studies have shown we can recall known disease-gene associations from OMIM using just cross-species phenotype comparisons with high specificity and sensitivity (area under curve of 0.85 from Receiver Operator Characteristic [ROC] analysis). This result, as well as showing our semantic comparison methodology works well, indicates that mouse phenotypes show a good match to the human clinical phenotypes for the majority of Mendelian diseases.

## Results

### PHIVE: An algorithm for cross-species phenotype analysis in whole-exome candidate gene prioritization

**Figure 1.**
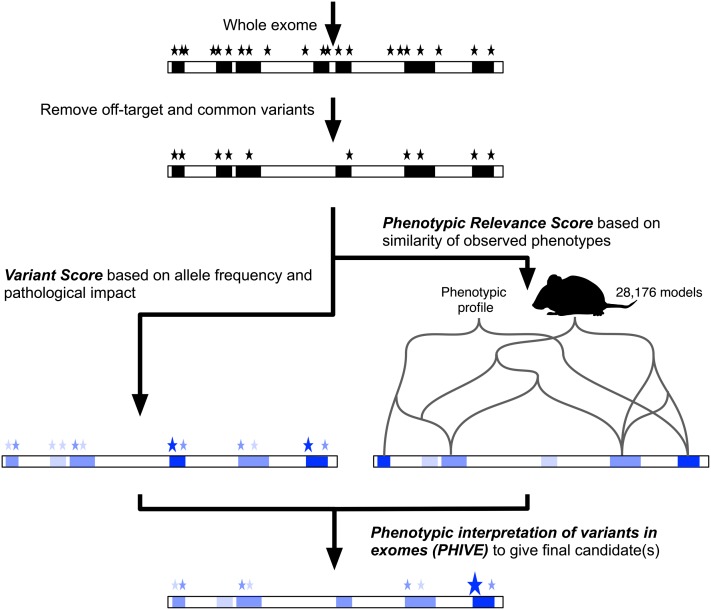
Exomiser filters a whole-exome data set by removing off-target, common, and synonymous variants from further consideration and evaluates the remaining variants based on the predicted pathogenicity and minor allele frequency (*variant score*). Optionally, an assumed mode of inheritance is used to further filter genes with variants present in a pattern compatible with the assumed mode of inheritance (e.g., homozygous or compound heterozygous for autosomal recessive). These genes are then assigned a *phenotypic relevance score* based on comparison with 28,176 mouse models with mutations in 9043 genes (7270 protein coding). The final ranking is calculated as the sum of the individual scores to yield the PHIVE score.

**Figure 2.**
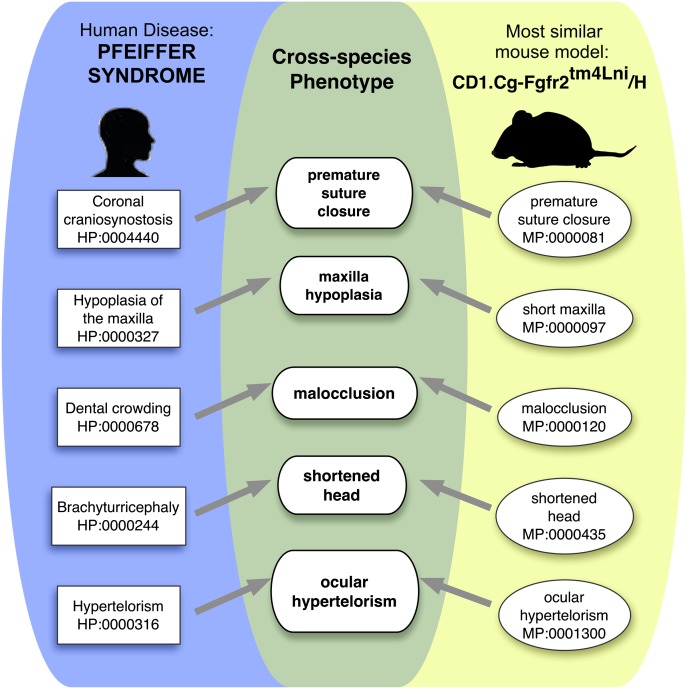
Phenotype matching algorithm. The user enters a human phenotype, either as an OMIM disease or as a list of HPO terms. All genes with variants that survive the initial filtering steps are then screened for mouse models with phenotypic similarity to the human disease. Similarity is calculated based on the semantic similarity of individual phenotypic features as described previously (Smedley et al. 2013).

To address the shortcomings of purely variant-based prioritization of WES data, we developed PHIVE (PHenotypic Interpretation of Variants in Exomes), an algorithm that first filters variants according to rarity, location in or adjacent to an exon, and compatibility with the expected mode of inheritance, and then ranks all remaining genes with identified variants according to the combination of *variant score* (frequency and pathogenicity of the variant[s]) and the *phenotypic relevance score*. In essence, our method searches for a phenotypically relevant gene that also has deleterious exome sequence variants, taking advantage of the voluminous data available for model organisms. Figures 1 and 2 summarize our procedure, and further details are given in the Methods section.

We have implemented our algorithm and made it freely available as the Exomiser Server (http://www.sanger.ac.uk/resources/databases/exomiser). Users upload their WES file in variant call format (VCF) and enter either the name of an OMIM disease, representing an established phenotypic profile for a Mendelian disease, or a set of clinical phenotypes encoded as HPO terms. Variants are then filtered according to optional, user-set parameters (e.g., variant call quality, minor allele frequency, inheritance model, removal of all nonpathogenic variants) and genes ranked according to the PHIVE score.

### PHIVE improves identification of correct disease gene in simulations on 28,516 known disease-causing mutations

To evaluate the expected performance of PHIVE, we developed a simulation strategy based on 28,516 known disease-causing mutations from the Human Gene Mutation Database (Stenson et al. 2008) associated with 936 genes and 869 diseases. We used 1092 WES files generated from “normal,” unaffected individuals by the 1000 Genomes Project Consortium (2012), and randomly added single disease-causing mutations to generate 100,000 simulated WES data sets per analysis. For autosomal dominant diseases, one heterozygous mutation was added; for autosomal recessive diseases, either one homozygous mutation was added or two heterozygous mutations in the same gene to represent the compound heterozygous model. Results were evaluated using ROC analysis, precision-recall plots, and by calculating the number of times the correct gene was ranked in first place by using the variant score alone, the phenotype relevance score alone, or their combination with PHIVE.

**Figure 3.**
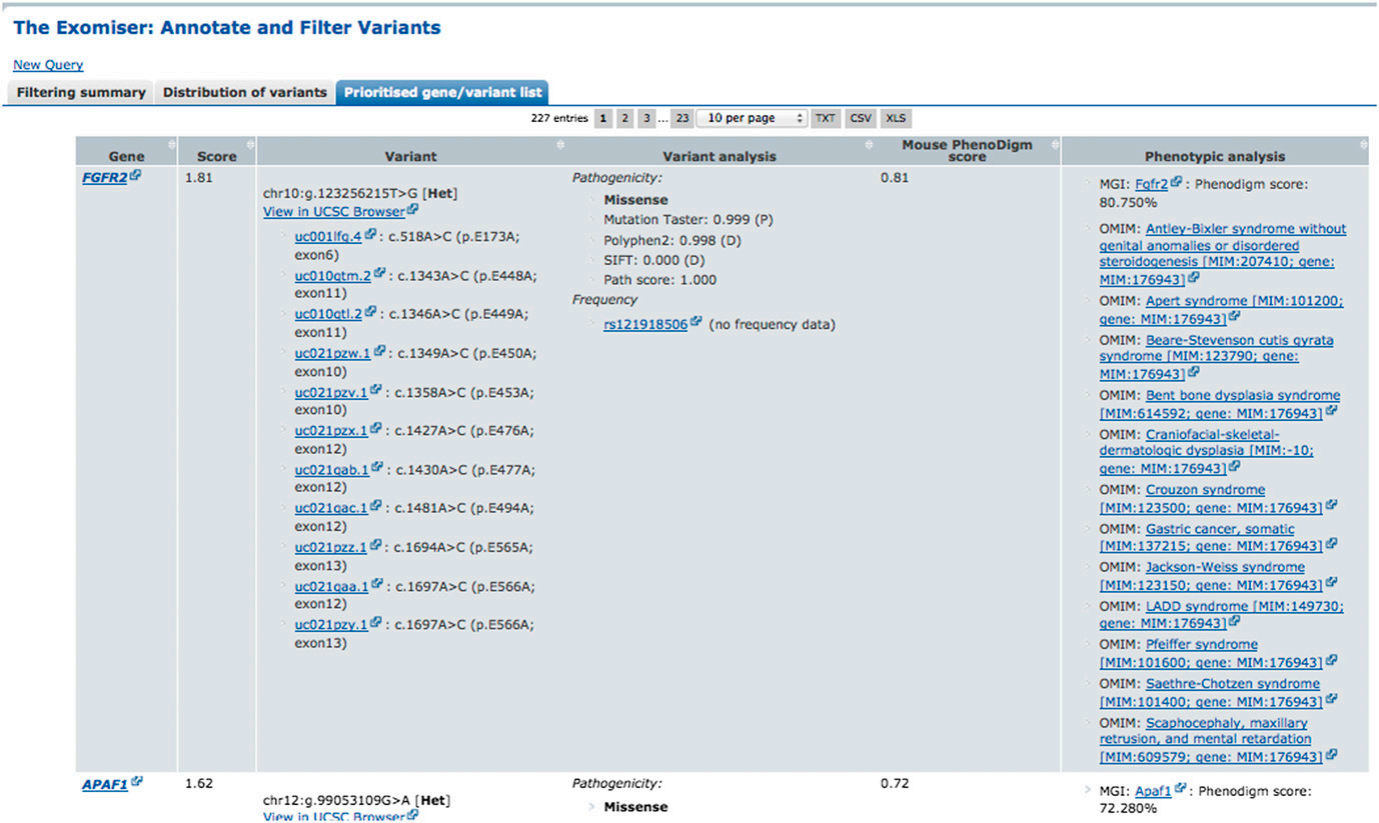
Exomiser querying of an exome containing a known chr10:g.123256215T*>*G heterozygous mutation associated with Pfeiffer syndrome (MIM:101600), an autosomal dominant Mendelian disease. The tab “Prioritised gene/variant list” shows the PHIVE prioritization of the 308 genes remaining after filtering of the original 8388 (details in Filtering summary table). The fully annotated variants associated with each gene, including pathogenicity and minor allele frequency, are shown along with the phenotypic relevance score from PhenoDigm and links out to any known phenotypic annotation from MGI/MGP or OMIM. The known variant is the top hit and annotated as a pathogenic, Glu to Ala missense coding change in *FGFR2*.

An example of our simulation approach is shown in Fig 3, in which the p.E173A mutation in the *FGFR*2 gene associated with Pfeiffer syndrome (MIM:101600) was added into a normal exome VCF file. Then, the clinical manifestations of Pfeiffer syndrome encoded as HPO terms were used to search among mouse models with mutations of genes with rare variants predicted to be pathogenic in this VCF file. Since the clinical manifestation of the FGFR2 mouse (CD1.Cg-Fgfr2^tm4Lni/H^) (Eswarakumar et al. 2004) displayed the highest degree of similarity, and the mutation was judged to be potentially pathogenic, the *FGFR*2 gene was listed as the top match by the PHIVE score.

Overall, our method ranked the correct gene as the top-scoring hit in 83% of exomes out of an average of 37 post-filtering candidate genes (minor allele frequency >1%, synonymous and off-target variants removed) under an Autosomal recessive (AR) model (Supplemental Table 1). Under an Autosomal dominant (AD) model, 66% of exomes had the correct gene as the top-scoring hit out of an average of 379 post-filtering candidate genes. This compares to 28% (AD) or 77% (AR) when using the variant-based scoring alone. Supplemental Figure 1 shows the corresponding precision/recall comparisons for autosomal recessive genes. The PHIVE score shows an improvement of between 1.1- and 2.4-fold in the percentage of candidate genes correctly ranked in first place compared to just using pathogenicity and frequency data. The overall area under the ROC curve was >95% (Supplemental Table 1).

**Figure 4.**
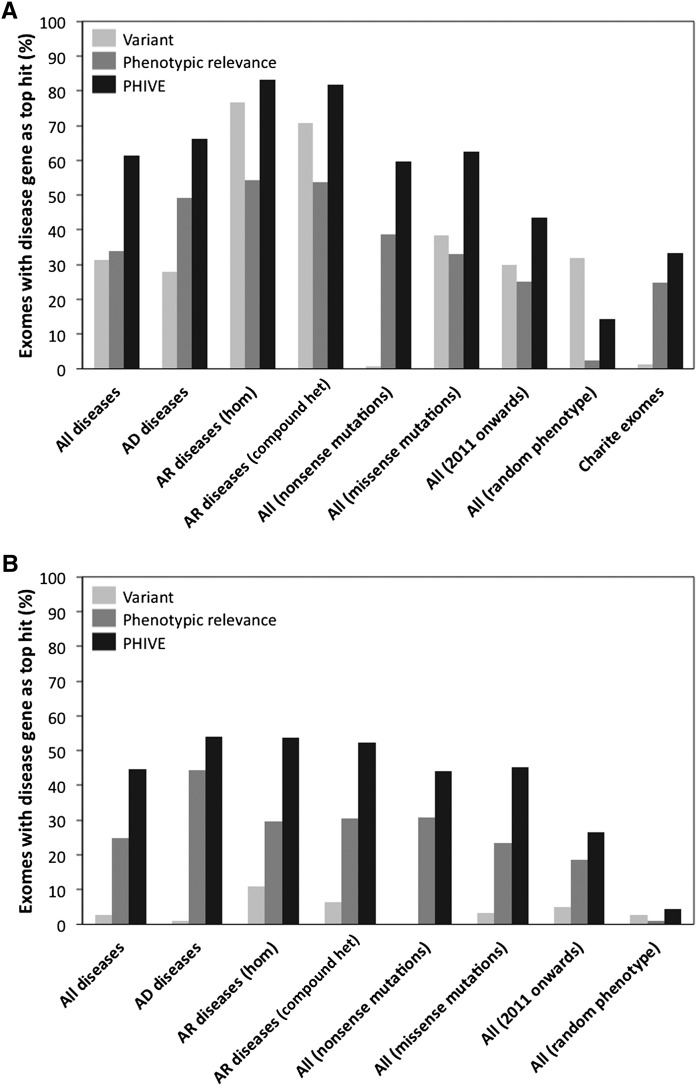
Comparison of different Exomiser filtering and prioritization strategies, including frequency data from either the ESP and the 1000 Genomes Project (*A*), or only ESP (*B*) to remove any potential bias due to the noncausative variants also coming from the 1000 Genomes Project. The first four groups of results show filtering of exomes (mean genes before filtering = 8388) by (1) removal of common, synonymous, and noncoding variants (mean genes after filtering = 400; 98.1% of disease variants retained) for *All* diseases, (2) further restriction to those compatible with *Autosomal dominant* (mean genes after filtering = 379; 98.5% of disease variants retained), or (3) *Autosomal recessive* inheritance by either homozygous or compound heterozygous mutation (mean genes after filtering = 37; 97.8% of disease variants retained). The performance for all diseases is also broken down into nonsense and missense mutations. In addition, we show the performance for all diseases in which the associated gene was discovered in 2011 or 2012 and the performance in which a random set of disease phenotype annotations were used rather than those of the disease being tested. Finally, the performance when adding known disease mutations to 144 exome samples from our own center rather than the 1000 Genomes Project exomes is shown. The bars show the percentage of times in which the true disease gene was assigned the top ranking match in 100,000 simulated WES data sets per analysis after prioritization based on the *PHIVE score*, *variant score*, and *phenotypic relevance score*.

Fig 4 shows the performance of the variant, phenotypic relevance, and PHIVE scores under various simulation conditions. For the 1000 Genomes Project simulations, the PHIVE score showed a substantial improvement over simply using pathogenicity and frequency data when not applying an inheritance model or under autosomal dominant inheritance and a moderate improvement under the autosomal recessive model. In the case of the latter, exome filtering already reduces the number of candidate genes to between 17 and 84, so the task of identifying the causative gene is simplified, even when just using the variant score. A control in which a randomly chosen disease (set of clinical phenotypes) was used instead of the disease associated with the added mutation, clearly shows the importance of the phenotype matching to the PHIVE score performance.

We also assessed the performance on the two major mutation types represented by known disease gene variation in HGMD: nonsense and missense. The performance for missense mutations is much better for the variant score and moderately better for the overall PHIVE score compared to that for nonsense mutations. This is not surprising as an average exome contains roughly 50 nonsense mutations that cannot be further discriminated by pathogenicity prediction algorithms, so variant-based prioritization performs poorly. Note that we chose a conservative pathogenicity score of 0.95 for nonsense and frameshift variants rather than 1.0 because of the observation that premature truncation codon (PTC) variants are not always pathogenic. Often, such PTC variants are located near the 3′ terminus of the affected gene. For instance, certain nonsense mutations in *ASXL3* are associated with syndromic intellectual disability, but other nonsense variants near the 3′ terminus are apparently neutral variants (Bainbridge et al. 2013). On the other hand, variants in the very 3′ region of genes can be pathogenic, and occasionally even cause different clinical diseases (Graul-Neumann et al. 2010). We therefore performed benchmarking using different pathogenicity scores for nonsense mutations and found the currently used value of 0.95 optimized our performance. Using a higher value, or always considering nonsense mutations as more pathogenic than missense, typically results in some of the roughly 50 nonsense mutations per normal exome scoring higher overall than the real, associated mutation with a consequent drop in our performance. Using a lower value results in real, associated nonsense mutations being missed and again drops our performance.

One potential criticism of our benchmarking is that the mouse models we are using for our comparisons may have been phenotyped in response to the discovery of a new disease-gene association. This could lead to an artificial improvement in performance compared to the real-life use case in which a novel disease-gene association is being assessed using existing mouse data. To test this, we ran another simulation in which we only used HGMD disease gene variants discovered in 2011 and 2012 that are less likely to have been extensively studied in a mouse model system. The phenotypic relevance and PHIVE score performance was reduced, which could be due to the reasons described above, but there was still a substantial improvement over variant-score prioritization.

The 1000 Genomes Project exomes tend to be more conservatively called than other variant calling pipelines, so to test the performance on exomes from other projects we reran the simulations on a set of 144 exomes generated at our own center. These VCF files, which were not prefiltered for on-target variants, contain many more variants (137,146–231,623 compared to 24,162–42,157 for the 1000 Genomes Project Consortium exomes). Performance was reduced, as may be expected with so many more false positive calls, particularly for the variant-score based prioritization in which there was a 15-fold drop in the number of exomes with the correct gene as the top hit. In contrast, the PHIVE score only showed a 1.8-fold drop in performance. This suggests that our combined PHIVE approach could be even more powerful relative to variant-based methods for real-life disease exome sequencing projects.

Another reason why the 1000 Genomes Project based simulations may perform better than real-life use cases is the fact that Exomiser uses frequency data from the Exome Server Project (ESP) and the 1000 Genomes Project for filtering and prioritization so all variants will have this data available. For in-house projects, there is a reasonable chance that a called variant has no frequency data in the ESP and the 1000 Genomes Project combined data set. To test what influence this may have had on our results, we reran the 1000 Genomes Project simulations but only using frequency data from the ESP project. As for our in-house exome simulations experiment, the variant-score-based performance showed a marked decrease (up to 10-fold), whereas the PHIVE based performance only decreased 1.3-fold when no inheritance model was used. This again points to the power of using phenotype-based comparisons for exome sequencing projects in which many of the called variants will have no frequency data in public data sets.

## Discussion

The field of computational disease-gene prioritization first came to prominence roughly a decade ago with the goal of pinpointing the most promising candidate genes within a larger multigene locus identified by positional genetic studies. A number of bioinformatic methods were developed to integrate complex and heterogenous data sets including expression data, genetic sequences, functional annotations, protein–protein interaction networks, and the medical literature. Many of the prioritization methods return a ranked list of genes that provide investigators clues about those genes most likely to reward closer investigation (Moreau and Tranchevent 2012; Bromberg 2013). Whole-exome sequencing, unlike linkage analysis, has the ability to identify causal variants directly. However, the diagnostic yield reported for large-scale WES studies has generally been substantially <50% (de Ligt et al. 2012).

Most approaches to the analysis of WES data have been filter-based, whereby variants are checked for novelty or rarity, predicted functionality (e.g., nonsynonymous variants at conserved sites), and sharing among affected individuals. A number of frameworks have been developed to improve upon the performance of filter-based prioritization. VAAST employs a number of filter steps followed by a likelihood ratio test that incorporates both amino acid substitution frequencies and allele frequencies to prioritize candidate genes on the basis of SNVs present in those genes in cases and controls (Yandell et al. 2011). If several families are available for analysis, rare variant burden tests have been applied with weighting of the variants by characteristics, including predicted pathogenicity or de novo status (Ionita-Laza et al. 2011). Additional filter criteria resulting from linkage analysis (Smith et al. 2011), pedigree analysis (Sincan et al. 2012), and inference of identical-by-descent regions (Rödelsperger et al. 2011) may be helpful in certain cases. A number of software tools are now available that allow the integrated analysis of WES data according to sequence-based filtering with functional annotations of the remaining candidate genes (Ge et al. 2011; Li et al. 2012; Sifrim et al. 2012; Teer et al. 2012; Zhang et al. 2013). While this manuscript was under review, two promising complementary approaches toward prioritizing variants in exome and genome sequencing were published. The eXtasy algorithm takes into account the predicted variant pathogenicity, haploinsufficiency predictions of the affected gene, and the similarity of the given gene to other genes associated with a user-supplied phenotype (Sifrim et al. 2013). eXtasy makes use of HPO terms and mappings (Köhler et al. 2009) to define training sets of genes that are used to seed prioritization by genomic data fusion with the previously described Endeavour algorithm (Aerts et al. 2006). Another approach was developed to prioritize potential disease-causing variants in noncoding sequences. The authors examined noncoding sequence regions showing evidence of constraint within the human population as evidenced by a high fraction of rare variants. By looking for such a signature of constraint within various genomic categories, such as DNase I-hypersensitive sites and transcription factor binding sequences (TFBS), ∼0.4% of the genome could be defined as “sensitive,” with these regions showing a number of interesting characteristics, such as enrichment for inherited disease causing mutations, some of which were found to disrupt a predicted TFBS. The methodology thus represents a powerful approach toward the prioritization of variants in noncoding regions (Khurana et al. 2013). PHIVE is unique among currently available exome prioritization algorithms in its use of semantic cross-species analysis to flag genes associated with phenotypes in mouse models that resemble the clinical characteristics of the patient being investigated by exome sequencing. The good performance of our method as well as eXtasy and the integrative approach to noncoding variants mentioned above, do suggest that some combination of clinical or biological aspects can greatly improve the performance of sequence-based exome analysis. It may be useful for future work to combine aspects of these algorithms to further improve performance.

**Figure 5.**
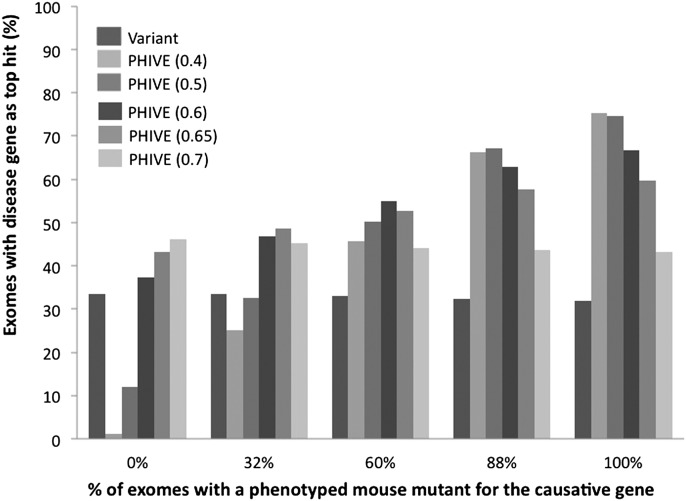
Comparison of different default *phenotypic relevance scores* for variants where no phenotyped mouse model exists for the gene containing the variant. The individual groups show the results after filtering to remove common, synonymous, and noncoding variants for exomes in which either 0, 32%, 60%, 88%, or 100% of the simulated exomes have a causative variant with mouse phenotype data for the orthologous gene. Thirty-two percent represents the current coverage of human protein-coding genes by phenotype data for the mouse ortholog. Eighty-eight percent represents the phenotypic coverage of disease-associated genes from the HGMD data set used throughout our studies. The bars show the percentage of times in which the true disease gene was assigned the top scoring match in 100,000 simulated WES data sets per analysis after prioritization based on either the *variant score* or *PHIVE score* using default *phenotypic relevance scores* of 0.4, 0.5, 0.6, 0.65, or 0.7.

In this work, we have designed a prioritization approach for WES data that searches for candidate genes within the wealth of phenotypic data on genetically modified mice. Our results show that PHIVE exhibits excellent performance for identifying the correct candidate gene for which phenotype data are available from a mouse model with a mutation in the corresponding gene. One obvious criticism of our approach is that if a mouse mutant does not exist for the disrupted gene, then our method may not be valid. To counter against this, we used a default phenotype score when no data were available and ran optimization simulations to choose a value in which the performance of PHIVE for exomes, where the causative gene has no mouse phenotype data was equivalent to that using variant-based methods (Fig. 5).

As would be expected, increasing the default score decreases performance when there is a mouse model for the causative gene but improves it when there is no phenotype data. The optimal value to use depends on how likely it is that the exome being analyzed will have a mouse model for the causative gene. From the current coverage of mouse protein-coding genes (7270 genes in the Exomiser database compared to 22,709 protein-coding genes annotated in Ensembl), one may predict a new disease exome analysis to have a 32% chance of having a mouse model for the causative mutation. On the other hand, 88% of the HGMD mutations we analyzed had mouse phenotype data for the gene in question, although there will clearly be some bias here in that some mouse models were created to study the disease mechanism after discovery of the causative gene (69.0% of the HGMD disease-associated variants had a mouse mutant of the gene that MGI already described as a model of the disease in question). However, hub genes in protein interaction networks are more likely to be essential nondisease genes, while the edge genes are more likely to be involved in diseases. Hence, the distribution of disease genes across the interactome is not even, increasing the probability that a newly discovered disease-gene association involves a gene already associated with another disease. Based on this, we predict that the real chance that a newly discovered disease association will involve a gene with existing mouse phenotype data will lie somewhere between 32% and 88%.

We settled on a final default of 0.6 for the phenotypic relevance score. At this level, even if there is no mouse model for the causative gene, the overall performance is equivalent to using the variant-based methods. It also optimizes performance where 60% of the exomes have a mouse mutant, which is likely to be close to the real coverage as discussed above. There is a drop in performance in cases in which the causative gene does have a mouse model compared to using a lower default score, but the gain in performance over the variant-based method is still substantial.

Data on the remaining mouse protein-coding genes will rapidly become available over the next decade through the efforts of the IMPC. Although the IMPC will only be performing a set battery of tests on each mouse line, most of the major disease areas are covered, although obviously not in the level of detail of a disease-focused project. To partially address this, several new grants have recently been awarded to characterize IMPC mice in the areas of developmental biology, immunology, and bone, and further projects are expected to follow soon. In addition, the IMPC actively encourages other groups to order mice, at a fraction of the cost of generating a new knockout, to perform additional, detailed phenotyping. To date, some 200 mouse lines have been ordered from the Sanger MGP, which is one of the major partners in the IMPC; and these mice were investigated and published, making extra phenotype data publicly available.

Furthermore, the use of phenotypes from the multitude of other model systems will not only validate these results but also potentially complement cases in which no mouse phenotype is available. Use of data from different model systems lends the advantage of inclusion of different types of phenotypes that are the focus in the different systems. To this end, Exomiser will be integrated as part of the Monarch Initiative (http://monarchinitiative.org) suite of tools to enable use of other model systems data in this context. In the meantime, we will seek to improve our performance where mouse phenotype data is missing, by inclusion of protein–protein interaction networks, coexpression, and other model organism data in future releases of Exomiser.

Obviously, as well as a good breadth and depth of mouse phenotype data, Exomiser will perform best where the human phenotypes are well defined, and we can only encourage physicians and clinical scientists to pay close attention to capturing the phenotype in a careful and comprehensive way (see Robinson 2012). However, the type of ontological similarity approach we adopt is robust in terms of adapting to minor errors in annotation, including imprecision, omissions, and noise (Köhler et al. 2009). The OMIM annotations that we used in our simulations contain a range of clinical phenotypes from those that are very detailed and specific to those that are nonspecific (e.g., nonsyndromic hearing loss, which is present in almost 100 syndromes). Therefore, our approach might prove useful even when only incomplete or imprecise phenotypic data is available for a sample.

In summary, the Exomiser provides a simple and highly effective way of prioritizing human candidate genes based on mouse phenotype comparisons alongside existing measures such as pathogenicity and minor allele frequency. Our results clearly show the value of comprehensive phenotypic data for computational analysis in translational bioinformatics. The approach is applicable to any disease exome sequencing project and, in particular, large-scale projects that are systematically annotating the samples to be sequenced using HPO.

## Methods

### Data sources

Information concerning population frequency of variants was derived from dbSNP (Sherry et al. 2001) and from the Exome Variant Server (NHLBI GO Exome Sequencing Project 2013, http://evs.gs.washington.edu/EVS/); and for this work, the maximum population frequency of a variant was taken to be its maximal reported frequency in any data source. For the dbSNP data, only the reported frequencies from the phase I 1000 Genomes Project variants were included. Information concerning predicted pathogenicity from SIFT (Ng and Henikoff 2002), PolyPhen-2 (Adzhubei et al. 2010), and MutationTaster (Schwarz et al. 2010) were extracted from dbNSFP (Liu et al. 2011). Links between genes and Mendelian diseases were extracted from data of the Online Mendelian Inheritance in Man resource (Amberger et al. 2011).

### Inheritance model filtering

The autosomal dominant filtering was performed by excluding any genes (and their variants) that did have at least one heterozygous variant that had passed all the previous filtering steps. Autosomal recessive filtering removes any genes where there is not at least one homozygous variant or two or more heterozygous variants that had passed the previous steps. X-recessive filtering requires a homozygous variant on a chromosome X located gene (because hemizygous variants on the X chromosome are called as homozygous in male samples).

### Phenotype ontologies

Phenotypic annotations to human diseases as listed in the OMIM database were extracted from the Human Phenotype Ontology (HPO) resource (Robinson et al. 2008). Mammalian Phenotype Ontology (MPO) annotations of mouse models (Smith et al. 2005), MGI asserted disease models, and OMIM human gene to MGI gene mappings were downloaded from the Mouse Genome Informatics ftp site (Bult et al. 2013) and the Sanger Mouse Portal (http://www.sanger.ac.uk/mouseportal).

### Variant annotation

Variants in the VCF files (which are defined using chromosomal coordinates) were annotated at transcript level using a Java implementation of ANNOVAR (Wang et al. 2010).

### Ranking candidate genes

Genes were ranked according to a combination of variant scores and phenotypic relevance scores as explained below. The variant score (*v*_*i*_) and phenotypic relevance score (*r*_*i*_) were used to calculate the PHIVE score for gene *i* as *g*_*i*_ = (*v*_i_ + *r*_*i*_)/2.

### Variant score

The variant score was defined to rank highly those variants that are both rare and predicted to be pathogenic. The estimated frequency of variants was derived from the 1000 Genomes Project Consortium data in dbSNP and from the Exome Server Project (ESP). Variants can be removed from further consideration if their population frequency exceeds a defined threshold (1% for some of the experiments described here). Any variants remaining after filtering are assigned a frequency factor as follows: Frequency factor = 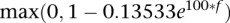 where 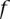 is the MAF between 0 and 1.

This results in values between 1 and 0 for MAF between 0 and 2%, with values >2% receiving a factor of 0. More stringent and lenient factoring was tested (see Supplemental Fig. 3), but factoring between 0 and 2% was found to optimize the performance of Exomiser on the known disease variants.

The predicted pathogenicity scores of SIFT, PolyPhen-2, and MutationTaster were normalized to lie between 0 (benign) and 1 (pathogenic). The scores of MutationTaster and PolyPhen-2 are such that the score ranges from 0 (benign) to 1 (maximally pathogenic). The SIFT score ranges from 1 (benign) to 0 (maximally pathogenic), and so it was transformed by *s′* = 1−*s*, where *s* is the original SIFT score. For missense variants, the pathogenicity score for each variant was then taken to be the maximum value of the MutationTaster, PolyPhen-2, and transformed SIFT scores. In some cases, no predictions were available from any of these three sources, and an arbitrary pathogenicity prediction of 0.6 was assigned. See Table 1 for other classes of variants.

**Table 1.**
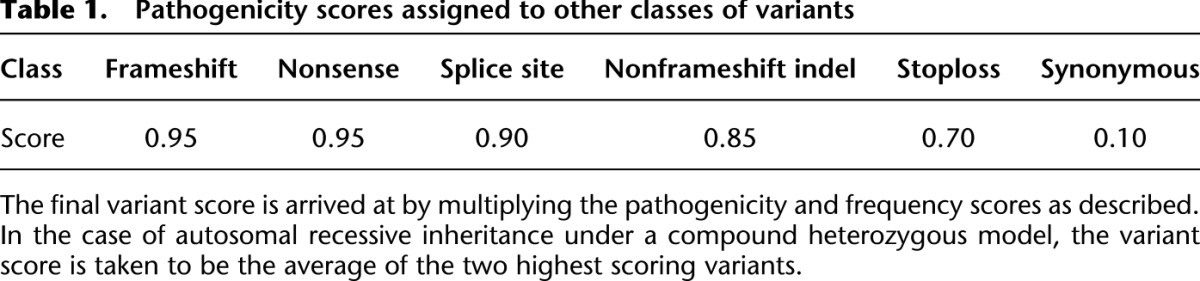
Pathogenicity scores assigned to other classes of variants

### Phenotypic relevance score

The phenotypic relevance score is calculated based on the semantic similarity of a human disease (the HPO annotations) and the phenotypic manifestations observed in a mouse model (the MPO annotation). OWLTools was used to calculate the phenotypic similarity between each of the HPO-annotated OMIM disease records and all 28,176 MPO-annotated mutant lines from MGI and the Sanger Mouse Genetics Project (Ayadi et al. 2012), resulting in a phenotypic relevance score for the corresponding mouse genes and their human orthologs. The pairwise comparisons were performed using OWL representations of the human and mouse phenotype annotations and a merged OWL file of the Phenotype and Trait Ontology (PATO), Uberon (Mungall et al. 2012), MPO and HPO and their logical definitions, as previously described (Doelken et al. 2013; Smedley et al. 2013). The logical definitions (Entity-Quality statements) are used to determine equivalent phenotypes in human and mouse where simple, lexical matching is not possible. For example, the HPO term *craniosynostosis* is defined by the entity *sutures* from Uberon and the quality *premature closure* from PATO. The MPO term *premature closure of the sutures* is similarity defined with *sutures* from Uberon and the *premature closure* from PATO, allowing a computational approach to detect that these two terms in different ontologies represent the same concept. Similar definitions are provided for other phenotypic features related to biological processes, small molecules, cell types, and anatomical structures.

The semantic matching approach allows similar but nonexact phenotypes to be detected and a score to be generated for how similar the two phenotypes being considered are and how specific the match is (generalized phenotypes that are seen in lots of diseases and mouse models receive a lower score). An overall similarity score between a disease (or set of clinical phenotypes) and a particular mouse model is obtained by averaging across all the pairwise comparisons between the individual clinical and mouse phenotypes. Thus, a high scoring mouse model represents similar phenotypes to many of the specific clinical phenotypes defining the disease. Finally, we take the phenotypic relevance score for a gene and disease (or set of clinical phenotypes) as the best score for any mouse model involving disruption of that gene.

### Validation of the PHIVE prioritization method

To validate our methodology, we developed a simulation strategy based on 28,516 known disease-causing mutations from the Human Gene Mutation Database (HGMD). These 28,516 mutations were selected on the basis of being assigned as a disease-causing, single-nucleotide mutation by HGMD and with HPO annotations available for the disease in question. We used 1092 whole-exome files (VCF) from the 1000 Genomes Project, and randomly added single disease-causing mutations for Mendelian diseases. The individual whole-exome files were extracted from the integrated call sets (Oct. 12, 2012 release at http://ftp.1000genomes.ebi.ac.uk/vol1/ftp/phase1/analysis_results/integrated_call_sets) using tabix (Li 2011) version 0.2.6 and VCFTools (Danecek et al. 2011) version 0.1.9.

For autosomal dominant diseases, one heterozygous mutation was added; and for autosomal recessive diseases, either one homozygous mutation or two heterozygous mutations were added to the 1000 Genomes Project VCF file. The phenotypic (HPO) annotations for the corresponding disease in OMIM (available from a long-term curation effort by ourselves at http://www.human-phenotype-ontology.org) were then compared to the MPO annotations for the 28,176 available mouse models, resulting in a phenotypic relevance score for the corresponding mouse genes and their human orthologs.

In all the analysis, an ordinal ranking method was used in which equal scoring genes are resolved arbitrarily but consistently by assigning a unique rank to each of the ties. In our case, we simply sort the equally scored genes alphabetically and assign the ranks. This corresponds to the real-life use case in which a researcher would have to take each of the equally scored top candidates and investigate each one by one for causality by further experimentation or for further candidacy by reviewing the literature/databases using their expert knowledge.

### Exomiser server

The methods described in this paper have been implemented in a freely accessible web server at http://www.sanger.ac.uk/resources/databases/exomiser.
